# Early and intermediate prognosis of intravenous thrombolytic therapy in acute ischemic stroke subtypes according to the causative classification of stroke system

**DOI:** 10.12669/pjms.291.2897

**Published:** 2013

**Authors:** Ali Pashapour, Abolfazl Atalu, Mehdi Farhoudi, Ali-Akbar Taheraghdam, Elyar Sadeghi Hokmabadi, Ehsan Sharifipour, Mehdi NajafiNeshli

**Affiliations:** 1 Ali Pashapour, Associate Professor, Departments of Neurology, Imam Reza Teaching Hospital, School of Medicine,Tabriz University of Medical Sciences, Tabriz, Iran.; 2 Abolfazl Atalu, Resident of Neurology, Neurosciences Research Center, Imam Reza Teaching Hospital, School of Medicine,Tabriz University of Medical Sciences, Tabriz, Iran.; 3 Mehdi Farhoudi, Associate Professor, Neuroscience Research Center, Imam Reza Teaching Hospital, School of Medicine,Tabriz University of Medical Sciences, Tabriz, Iran.; 4 Ali-Akbar Taheraghdam, Assistant Professor, Departments of Neurology, Resident of Neurology, Neurosciences Research Center, Imam Reza Teaching Hospital, School of Medicine,Tabriz University of Medical Sciences, Tabriz, Iran.; 5 Elyar Sadeghi Hokmabadi, Imam Reza Teaching Hospital, School of Medicine,Tabriz University of Medical Sciences, Tabriz, Iran.; 6 Ehsan Sharifipour, Imam Reza Teaching Hospital, School of Medicine,Tabriz University of Medical Sciences, Tabriz, Iran.; 7 Mehdi NajafiNeshli, Resident of Neurology, Neurosciences Research Center, Imam Reza Teaching Hospital, School of Medicine,Tabriz University of Medical Sciences, Tabriz, Iran.

**Keywords:** Ischemic stroke, Thrombolytic therapy, Etiology, Outcome, Ischemic Stroke Subtypes

## Abstract

***Objectives:*** Intravenous thrombolytic therapy has established acceptable results in treating ischemic stroke. However, there is little information on treatment outcome especially in different subtypes. The aim of current study was to evaluate early and intermediate prognosis in intravenous thrombolytic therapy for acute ischemic stroke subtypes.

***Methodology: ***Forty eligible patients (57.5% male with mean age of 63.18±13.49 years) with definite ischemic stroke who were admitted to emergency department of Imam Reza University Hospital, in the first 180 minutes after occurrence received recombinant tissue plasminogen activator. All investigation findings were recorded and stroke subtypes were determined according to the Causative Classification of Stroke System. Stroke severity forms including modified Rankin Scale (mRS) and National Institutes of Health Stroke Scale (NIHSS) scores were recorded for all patients in first, seven and 90 days after stroke and disease outcome was evaluated.

***Results:*** The etiology of stroke was large artery atherosclerosis in 20%, cardio-aortic embolism in 45%, small artery occlusion in 17.5% and undetermined causes in 17.5%. NIHSS and mRS scores were significantly improved during time (P < 0.001 in both cases). Three months mortality rate was 25%. Among the etiologies, patients with small artery occlusion and then cardio-aortic embolism had lower NIHSS score at arrival (P = 0.04). Caplan-meier analysis showed that age, sex and symptom to needle time could predict disease outcome.

***Conclusion:*** Intravenous thrombolytic therapy is accompanied by good early and intermediate outcome in most patients with ischemic stroke. Small artery occlusion subtype had less disease severity and higher improvement.

## Introduction

 Stroke is the third leading cause of death worldwide and is one of the most important factors of disability in different countries.^1^^-^^3^ Although stroke appears to be a disease of the elderly, but one third of patients have less than 65 years of age.^4^ Among them, 50 to 70 percent of patients get back their primary function, 15 to 30 percent remain incapable for ever and 20 percent are in need of hospital care for three months.^5^ With better understanding of the pathophysiology of nerve damage and introduction of new drugs, treatment regimen includes anticoagulation and thrombolysis drugs besides invasive interventions.^6^

 Recombinant tissue plasminogen activator (rtPA) in the treatment of cerebral ischemic stroke has been confirmed by American Food and Drug Administration (FDA) in 1996.^7^ In America, this drug causes symptoms improvement of stroke in more than 50% of cases with minimal serious complications.^8^ However, only 3 to 8.5% of patients were eligible for rtPA.^9^ Several studies about cost - effectiveness of this drug compared with the high cost and potential side effects of this drug have been performed. Results show that use of rtPA has increased for ischemic stroke treatment due to reduction in patient morbidity and hospitalization time and no need to give long-term treatment, also saving millions of dollars.^10^

 Studies that evaluate the prognosis of etiologic subtypes in patients receiving intravenous thrombolytic therapy are limited and these studies mainly have reported complications following drug consumption.^11^^-^^13^ The aim of this study was to evaluate the prognosis of intravenous thrombolytic therapy in ischemic stroke subtypes to investigate the early (7 days) and intermediate (3 months) effect of desired drug.

## Methodology

 This study was performed by a stroke team and rtPA was administrated in the treatment of acute ischemic stroke as a prospective descriptive-analytical study. The study population consisted of 40 patients who were hospitalized at Imam Reza Hospital Emergency during September 2011 to March 2012 with symptoms of suspected stroke and in 180 minutes of the onset of their clinical symptoms. This study was database of a large trial that is being conducted and named as Tabriz Thrombolytic Therapy for Acute Ischemic Stroke (T3-AIS).

**Fig.1:**
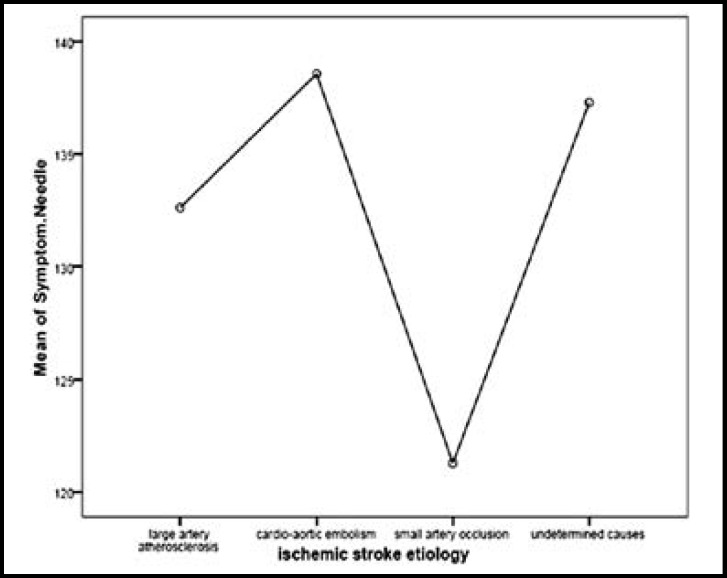
Interval of symptoms presentation until TPA administration in various etiologies

 Inclusion criteria was patients under the Cincinnati Pre-hospital Stroke Scale with at least one of three symbols including unilateral facial weakness, unilateral upper extremity weakness (Arm drift) and abnormal speech, and less than 180 minutes from the onset of their symptoms. Of all patients in the triage room laboratory sampling and brain CT scan were performed. With detailed history taking and physical examination contraindication of rtPA administration from the form shared guideline of American Heart Association and American Stroke Association (year 2010)^14^ (that indications and contraindications of rtPA administration has determined) was evaluated and forms were completed. With definite diagnosis of ischemic stroke and in the absence of rtPA administration contraindications, patients were admitted to the neurology ICU, written consent was obtained from patient’s relatives.

 Clinical Assessment Forms of stroke using National Institutes of Health Stroke Scale (NIHSS) and modified Rankin Scale (mRS) were completed for all patients and in the next step was attempted to administer rtPA. Patients after this emergency phase were undergoing routine investigations according to case and to determine the etiology of the disease.

 All findings of medical history, physical examination, brain imaging, vascular imaging, cardiac investigations and other items were recorded. In accordance with the Causative Classification of Stroke (CCS)^15^ specific subtype of stroke was determined. The CCS is a questionnaire-style classification scheme for ischemic stroke. The automated algorithm reports the stroke subtype and a description of the classification rationale. It arranges the patient into 5 subgroups (large artery atherosclerosis, cardio-aortic embolism, small artery occlusion, other causes, and undetermined causes) and each subtype of stroke subdivided to evident, probable and possible. The undetermined group is divided into cryptogenic embolism, other cryptogenic, incomplete evaluation, and unclassified categories and NIHSS and mRS were determined for each patient on day 1, 7 and 90.

**Fig.2:**
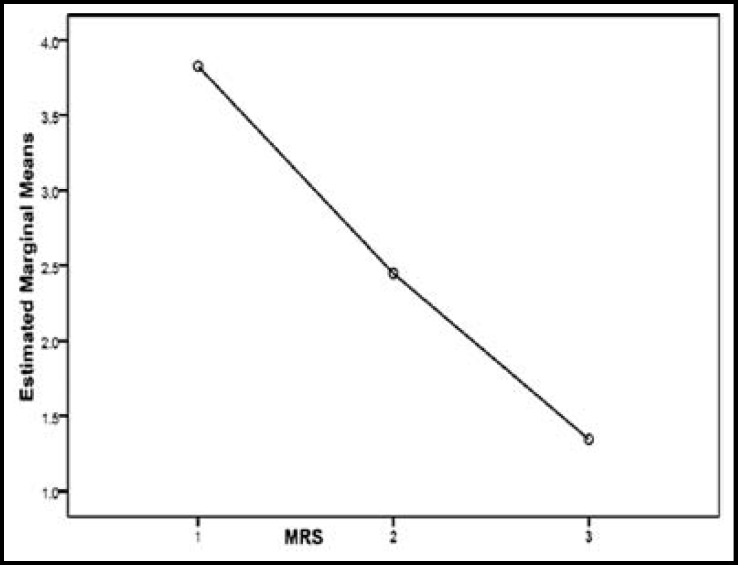
mRS scale changes over the 3 months

**Fig.3:**
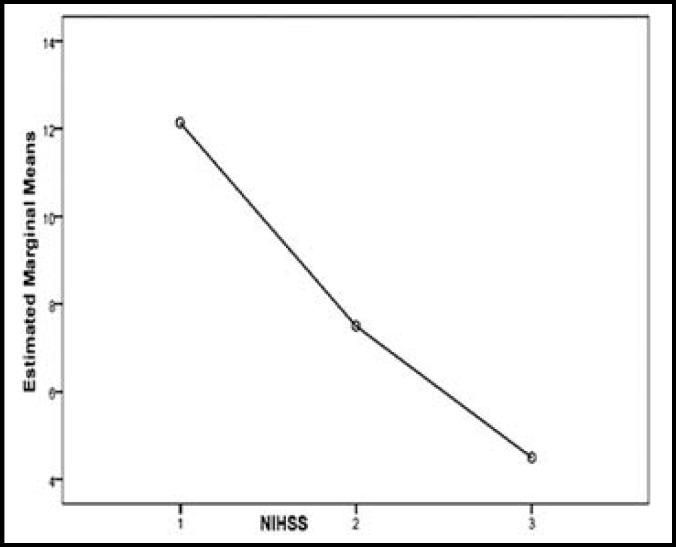
NIHSS scale changes over the 3 months

This study was designed as ongoing administration of thrombolytic therapy and in this regard, legal and ethical standards were considered.

***Statistical Analysis:*** Obtained data are expressed as mean ± standard deviation, frequency, percentage and SEM (if necessary). Data were analyzed by SPSS™ 17 software. Quantitative variables were compared by using Student T-test and one way ANOVA and multiple regressions and qualitative variables have been compared by using Chi-Square Test and Fisher’s Exact Test. Quintile and Cox regression analysis was used to determine the disease prognosis predicting factors. In all investigated cases, the results have been known statistically significant in case of P ≤ 0.05.

## Results

 In this study 40 patients with ischemic stroke underwent rtPA treatment with mean age of 63.18±13.49 (38 – 88 years) with a median of 67.50 year. The patients were 23 (57.5%) male and 17 (42.5%) female. Average systolic blood pressure was 149.18±31.05 mmHg and mean diastolic blood pressure was 88.08±14.16 mmHg.

**Fig.4:**
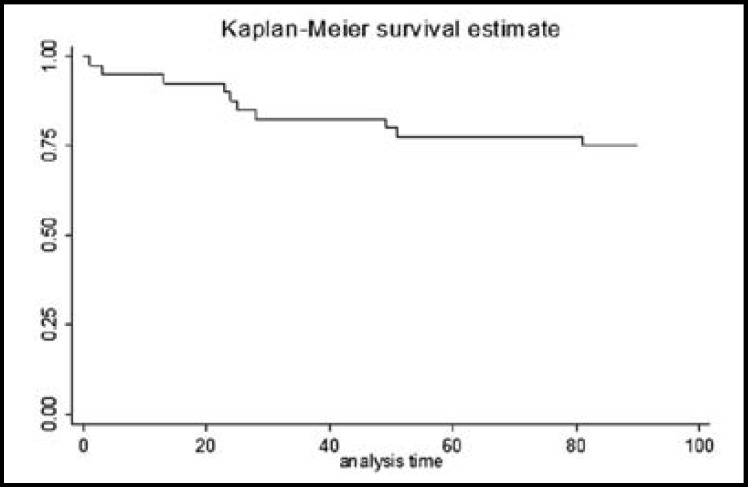
Kaplan-Meier curve of total patients survival

 As regards past medical or social history, 24 patients (60%) had HTN, 10 (25%) had Diabetes Mellitus, 9 (22.5%) were smokers, 5 (12.5%) had hyperlipidemia. AF was detected in 9 (22.5%), and 11 (27.5%) suffered from IHD. Embolus history was noted in 9 (22.5%) out of which 7 cases (17.5%) were ischemic strokes or TIA and in 2 cases (5%) was associated with systemic emboli. Mean NIHSS score on admission equaled 13.38±4.46 with a median of 13. The highest and the lowest scores were 3 and 27 respectively. NIHSS score in 17 patients (42.5%) was more than 15 or in other words had severe stroke on admission.

**Table-I:** NIHSS score at different stages in the various etiology of diseases based on mean; NIHSS: National Institutes of Health Stroke Scale

	*Large artery atherosclerosis*	*cardio aortic embolism*	*Occlusion of small vessels*	*Undetermined causes*	*P value*
Admission NIHSS	14.62 ± 5.85	6.56 ± 1.54	3.62 ± 1.37	14.14 ±6.38	0.04
Seventh day NIHSS	13.12 ± 8.40	10.50 ± 8.56	6.86 ± 2.13	9.43 ± 3.88	0.2
Third month NIHSS	7.29 ± 5.02	4.92 ± 1.65	1.00 ± 0.54	3.33 ± 1.25	0.15

**Note:** Data are presented as Mean ± SD

 On admission mRS score average was 4±1.25 with a median of 4. The highest and the lowest scores were 1 and 5 respectively. Using the CCS to determine etiologic subtype of stroke 8 (20%) large vessel atherosclerosis, 18 (45%) cardio-aortic embolus, 7 (17.5%) small vessel occlusion and 7 (17.5%) no known cause were involved. With the CSS subdivision, patients were placed in 9 subgroups as follows:7 (17.5%) documented large vessel atherosclerosis, 2 (5%) probable large vessel atherosclerosis, 9 (22.5%) documented cardio-aortic emboli, 2 (5%) probable cardio-aortic emboli, 7 (17.5%) possible cardio-aortic, 7 (17.5%) documented small vessel occlusion, 2 (5%) with obviously unknown factor, 3 (7.5%) with probable unknown cause, and 1 (2.5%) with possible cause.

**Fig.5:**
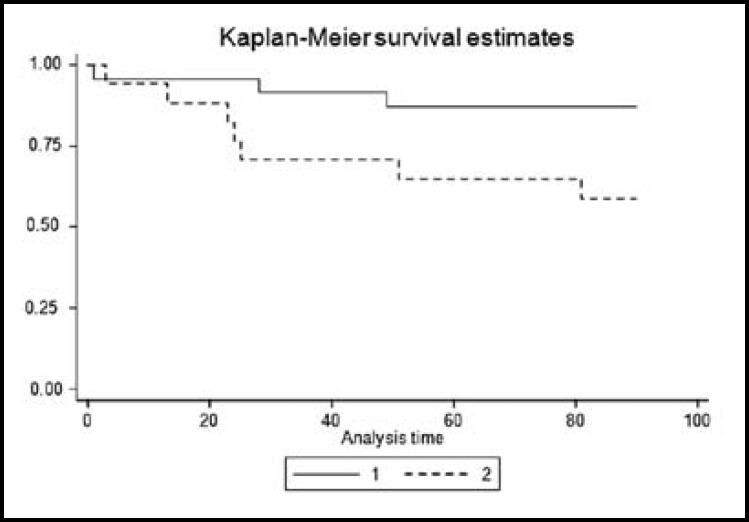
Kaplan-Meier curve of patients based on gender

 However in one instance, CSS software grouped one of the patients to probable large vessel atherosclerosis, probable cardio-aortic emboli, and unclassified unknown etiology and in another instance one case was classified both as evident cardio-aortic emboli and possibly unknown etiology; we considered them to be probable and evident respectively. Fig.1 demonstrates the period between onset of symptoms and rtPA administration in various etiologies. As seen the patients with small vessel occlusion sought medical care more immediately. No significant difference between groups were seen in this respect (P =0.69).

Fig. 2 and 3 show the trends for changes in mRS and NIHSS criteria during 3 months. As shown in the diagrams as time elapsed mRS and NIHSS scores decreased to a significant degree meaning recovery has occurred (P <0.001 in both cases). At the end of 3 months there were 10 deaths (25%) of which four (10%) were due to direct stroke and rtPA complication, while enough follow up data is not available about other six patients. Two death occurred in the first week and the other between week one and month three. Considering low sample size in this study, it was not possible to compare patients regarding CCS criteria; however patients were investigated as to the etiologies.

 Death at the end of three months had occurred in one case (12.5%) with large vessel atherosclerosis, in 6 (33.3%) of cases with cardio-aortic emboli, in 2 cases (28.6%) with small vessel occlusion and one (14.6%) of those having no specified etiology. It was not feasible to compare data statistically because of uneven distribution.

Table-I, shows NIHSS scores for different etiologies in different stages. Patients with small vessel occlusion and then cardio-aortic emboli have lower NIHSS scores or in fact less severe stroke (P =0.04). However in these two groups conditions worsened and in two other groups changed for better till day 7. At the end of three months the group with small vessel occlusion had lower NIHSS score and significant recovery; although no statistically significant difference exist between two groups.

Table-II, shows mRS scores for different etiologies in different stages. Cardio-aortic group had the highest mRS scores on admission. Following treatment decrease in MRS and recovery in small vessel occlusion on day 7 and after three month was higher; however no statistically significant difference was observed. Applying Quintile regression test was not able to identify predictors for prognosis based upon mRS, NIHSS. Hence in this respect Cox regression was applied in which the three variables: age, sex, and time between onset of symptoms and administration of rtPA were capable of yielding a meaningful survival prognosis. It should be noted that symptom to needle time and confidence interval for Hazard Ratio was wide due to scarcity of cases.

Fig. 4 and 5 demonstrate Kaplan-Meier analysis result for three-month survival in terms of age which was 1.19 ± 0.062 (P < 0.001, z = 3.34, CI: 1.07 – 1.32), gender was 22.30 ± 23.20 (P = 0.003, z = 2.98, CI: 2.90 – 171.33) and symptoms was 1.03 ± 0.01 (P = 0.04, z = 1.99, CI: 1.0004 – 1.06).

## Discussion

 Stroke leads to very high medical costs to healthcare systems^1^^,^^2^ and to reduce it, administration of rtPA in patients with stroke had come into attention. The minimum benefit of increasing the use of rtPA in the treatment of patients with ischemic stroke include rapid evacuation of hospital beds, reduction of need in and equipment reducing the burden of nursing care.^11^ However, lack of infrastructural facilities, expensive drugs, side effects of drug prescription and low golden time from onset of symptoms until the drug administration has limited the using of this medication.^11^^-^^13^

 Yip et al has concluded that even with slight increase in rtPA administration rate in Canada significant savings in Health Care System expenditures were obtained.^1^ In two other studies done by Coan et al increasing rtPA administration has been reiterated.^16^^,^^17^ However few studies have investigated the prognosis of patients after intravenous thrombolytic therapy according to the etiologolic subtypes.^11^^-^^13^^,^^18^^-^^20^ Imam Reza Teaching Hospital, a tertiary health center is annually visited by over 1000 ischemic stroke patients. Physicians and nurses are experienced in treatment, having capability for neuro imaging, consultation and brain surgery in a round the clock mode. In addition it has facilities of intensive care units, physiotherapy and nutrition consultations which has provided the infrastructure required for routine and systematic administration of rtPA by triage of ischemic stroke patients and setting up special team. Current study has investigated short and long-term prognosis of intravenous thrombolytic therapy within 3 hours in ischemic stroke subgroups of 40 patients admitted with stroke.

**Table-II:** mRS score at different stages in the various etiologies of diseases based on mean; mRS: Modified

	*Large artery atherosclerosis*	*cardio aortic embolism*	*Occlusion of small vessels*	*Undetermined causes*	*Quartile (25 – 75%)*	*P value*
Admission mRS	3.88 ± 1.8	4.38 ± 0.88	3.14 ± 1.34	4.14 ± 0.9	3.25	0.17
Seventh day mRS	3.12 ± 1.95	3.12 ± 1.74	1.71 ± 0.74	2.71 ± 1.89	3.75	0.4
Third month mRS	1.71 ± 1.6	1.58 ± 0.54	0.2 ± 0.2	1.17 ± 0.6	3	0.38

**Note:** Data are presented as Mean ± SD

 Complications associated with drug especially intracerebral hemorrhage (ICH) has deterred physicians from using it.^12^ We too encountered 17.5% ICH rate, four of which were symptomatic and resulted in death. In a study by Walters et al on 120 patients, 16 episodes of acute cerebral hemorrhage occurred, 5 of which were symptomatic.^21^ Studies have cited different figures for cerebral hemorrhage prevalence varying from 3-13%.^12^^,^^13^^,^^21^ This can be attributed to general health of patients studied in each group and presence or absence of diseases and underlying etiology in each etiology.

 This study has demonstrated significant recovery in mRS and NIHSS scores following treatment. 3-month mortality was 25% and severe morbidity occurred in 10% of patients. About 65% of patients had no or least morbidity according to mRS criteria. Figueroa- Reyes et al studied outcome of ischemic stroke following rtPA in 19 patients out of which 63% suffered no or least morbidity, 26% medium and 5% severe morbidity. One case died one day after thrombolysis.^22^

 In Walters et al survey over 3 month follow up period, 31% of patients enjoyed favorable results (MRS 0-1), 22% gained average results (MRS 2-3) and 21% had poor results (MRS 4-5) with 21% mortality.^21^ Discrepancy in mortality observed in various studies can be due to difference in sample sizes. Generally it can be noted that in all studies most of patients take benefit out of rtPA treatment and recover with sufficient restoration of functions. Key outcomes such as disability, death and recurrence following ischemic stroke differ between subgroups.^23^^,^^24^ In our survey etiologies were large vessel atherosclerosis in 20%, cardio-aortic emboli in 45%, and small vessel occlusion in 17.5%. Of all etiologies, small vessel occlusion followed by cardio-aortic emboli had lowest NIHSS scores. Patients suffering from small vessel occlusion visited the hospital earlier than others.

 This study also showed that recovery within three months in patients with small vessel occlusion was higher than others. After three months death occurred in 12.5% of patients with large vessel atherosclerosis, 33.3% of patients with cardio-aortic emboli, 28.6% with small vessel occlusion and 14.3% of episodes had no known etiology. Other studies have shown small vessel involvement and lacunar infarct to have better recovery and less recurrence.^25^^,^^26^ Severity of neurologic deficit in the beginning of the disease has been postulated as the most important factor in prognosis. Accordingly one study showed NIHSS over 17 and age over 70 to be the best one-year prognostic factors of poor outcome.^27^ However in our study despite high NIHSS scores and advanced ages in patients with poor prognosis, no significant difference was observed in this respect. We saw that age, sex and time between symptoms onset and drug administration were capable of forecasting disease outcome. Similarly Dharmasaroja et al have stated that age over 70, severe stroke (NIHSS ≥ 15) and Intracranial Hemorrhage (ICH) are conversely related to early recovery.^28^

## Conclusions

 Intravenous thrombolytic therapy is accompanied by favorable short and long-term results in patients with ischemic stroke. Symptoms are less severe and recovery is higher in patients with small vessel occlusion. Age, sex and time between symptoms onset and drug administration were capable of forecasting disease outcome. Considering findings of this study intravenous thrombolytic therapy can be recommended especially in milder episodes and small vessel occlusion subgroups. However due to recent administration of rtPA in our center and small sample size of our study it was not feasible to evaluate statistical difference between all subgroups. Conducting a study with larger sample size can yield better and more precise results.
